# A Novel Chemical-Space-Dependent Strategy for Compound Selection in Non-target LC-HRMS Method Development Using Physicochemical and Structural Data

**DOI:** 10.1021/acs.estlett.5c00759

**Published:** 2025-08-18

**Authors:** Lapo Renai, Viktoriia Turkina, Tobias Hulleman, Alexandros Nikolopoulos, Andrea F. G. Gargano, Elvio D. Amato, Massimo Del Bubba, Saer Samanipour

**Affiliations:** † Van’t Hoff Institute for Molecular Sciences (HIMS), 1234 University of Amsterdam, 1090 GD Amsterdam, The Netherlands; ‡ Queensland Alliance for Environmental Health Sciences (QAEHS), 20 Cornwall Street, Woolloongabba, QLD 4102, Australia; § ARC Training Centre for Hyphenated Analytical Separation Technologies (HyTECH), Queensland Alliance for Environmental Health Sciences (QAEHS), 20 Cornwall Street, Woolloongabba, QLD 4102, Australia; ∥ KWR Water Research Institute, Groningenhaven 7 3433 PE Nieuwegein, The Netherlands; ⊥ Department of Chemistry, 9300 University of Florence, Via della Lastruccia 3, 50019 Sesto Fiorentino, Florence, Italy; # UvA Data Science Center, University of Amsterdam, 1012 WP Amsterdam, The Netherlands

**Keywords:** Non-target Analysis, Chemical Space, Exposomics, Emerging Contaminants, Mobility, Ionization Efficiency, Liquid Chromatography, Mass Spectrometry

## Abstract

The virtual chemical space of substances, including emerging contaminants relevant to the environment and exposome, is rapidly expanding. Non-targeted analysis (NTA) by liquid chromatography–high-resolution mass spectrometry (LC-HRMS) is useful in measuring broad chemical space regions. Internal standards are typically used to optimize the selectivity and sensitivity of NTA LC-HRMS methods, assuming a linear relationship between structure and behavior across all analytes. However, this assumption fails for large, heterogeneous chemical spaces, narrowing measurable coverage to structurally similar compounds. We present a data-driven strategy for unbiased sampling of candidate structures for NTA LC-HRMS method development from extensive chemical spaces, such as the U.S. EPA’s CompTox (>1 million chemicals). The workflow maximizes physicochemical/structural diversity using precomputed PubChem descriptors (e.g., molecular weight, XLogP) and grants LC-HRMS compatibility thanks to predicted mobility and ionization efficiency from molecular fingerprints. The resulting measurable compound lists (MCLs) provide broad, heterogeneous coverage for NTA method development, validation, and boundary assessment. Applied to the CompTox space, the approach yielded MCLs with greater chemical coverage and broader predicted LC-HRMS applicability than conventional “watch list” contaminants, offering a robust framework for enhancing NTA’s measurable chemical space while preserving diversity.

## Graphical abstract

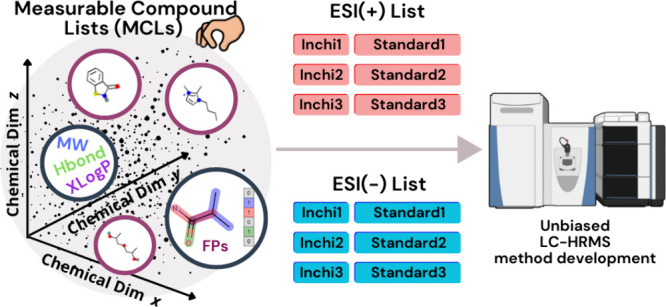

## 1 Introduction

The concept of chemical space has been introduced to depict the virtual regions occupied by all the existing and probable molecules according to their structural and physiochemical properties.^1,2^ There is no unified vision of the entire chemical universe, and even if one existed, its high dimensionality and continuous expansion would limit any practical applications. For instance, based on physicochemical, bioactivity, bioavailability, and toxicity descriptors, the virtual space of small organic molecules is estimated to contain over 10^60^ compounds.^3,4^ Consequently, a “practical” chemical (sub)­space is typically chosen based on the compounds present in the sample matrix being analyzed. For example, the exposome space of emerging contaminants that humans encounter throughout their lives is addressed when dealing with environment-relevant samples (e.g., water samples).^5,6^

In theory, the comprehensive measure of a selected chemical subspace, exposome included, is enabled by non-target analysis (NTA) approaches, relying on data generated via high-resolution mass spectrometry (HRMS) hyphenated with separation techniques such as liquid chromatography (LC).^7,8^ Yet, the measurable regions of a selected chemical space are limited to LC-HRMS-detectable compounds and depend on the quality of NTA study design,^4^ including sample preparation and chromatographic analysis.^6,9^

These analytical steps impact the selectivity and sensitivity of recorded signals, affecting the composition of measured space.^10,11^ To optimize selectivity (e.g., analyte recovery and peak capacity) and sensitivity (i.e., HRMS signal quality for precursor and fragmented ions), analytical NTA workflows use internal standards that represent the relevant chemical subspace.^12^ In exposome analysis, chemicals of environmental concern (CECs) included in the European monitoring and/or ENTACT initiative lists are often used.^13–16^ This strategy assumes linear relationships between properties (e.g., LogP and molecular weight) and the retention/ionization behaviors of internal standards relevant to the chemical subspace.^12^ While valid for targeted analysis of specific compounds, this assumption fails for NTA of a heterogeneous chemical subspace due to the complex (non-linear) nature of the chemicals and samples involved.^11,17^ As an example, Figure 1 depicts the distribution of ∼800,000 exposure-relevant chemicals from the CompTox database,^18^ highlighting overlaps between EU monitoring (*n* = 62) and ENTACT lists (*n* = 1019). While these subgroups show a linear trend, the overall CompTox distribution is irregular.

**Figure 1:**
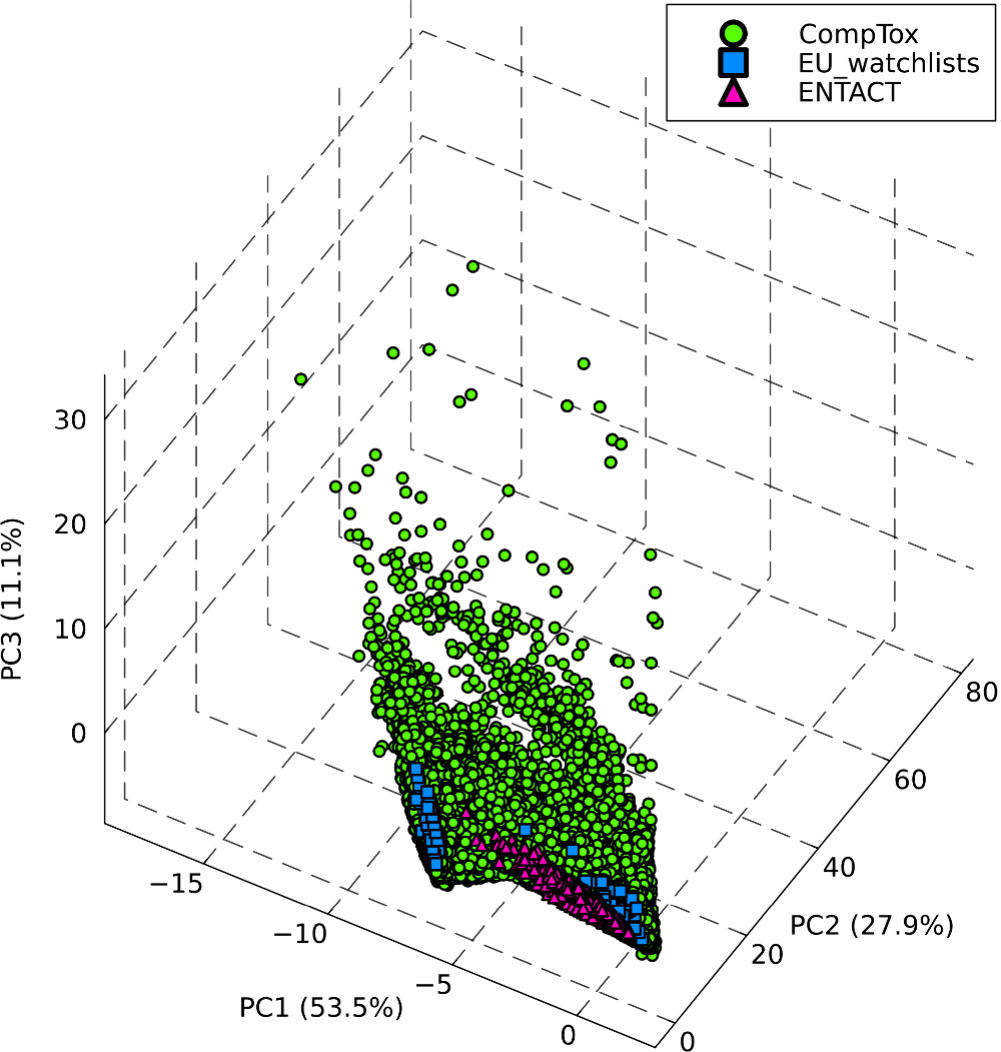
Score plot of the principal component analysis (PCA) on chemical structures within the CompTox database (*n* ≈ 800k) and the overlap with the shared compounds listed in European water monitoring program (*n* = 62) and the prioritized list of the ENTACT initiative (*n* = 1019). PCA was carried out on compounds’ physicochemical properties extracted from PubChem (e.g., molecular weight and XLogP) and six calculated elemental mass defects (e.g., CO, CCl, and CN), which were used as model variables. See Section S5 of the Supporting Information for a 2D plot.

Thus, the selection of a few internal standards with limited chemical coverage can bias NTA method development. This restriction means that, for LC-HRMS, only a small set of chemically similar compounds may meet the necessary detection and identification criteria, which limits the measured subspace and decreases the discovery rate within the chosen chemical space.^4,11^

To minimize such bias in NTA analysis, it is crucial to select standards that accurately represent the desired chemical subspace and are LC-HRMS compatible, as well as managing their quantity and costs.^19^ In this context, we present a data-driven and unbiased approach for the comprehensive and reproducible selection (or “sampling”) of chemicals within a selected chemical subspace (e.g., exposomics). This method aims to generate measurable compound lists (MCLs) that serve two primary purposes: (i) maximizing the measurable space and (ii) establishing the applicability and detection boundaries of the LC-HRMS NTA methods. We utilize the exposome chemical space, approximated by the CompTox database. Chemical selection incorporates precomputed physicochemical properties, including predictions of environmental mobility and ionization efficiency (IE) as descriptors to identify candidate substances relevant for environmental monitoring and compatible with LC-HRMS analysis.^20–23^

## 2 Materials and Methods

### 2.1 Workflow for MCL Sampling

The overall workflow for computing the chemical subspace and extracting MCLs starts from the conversion of CompTox dataset canonical SMILES into a set of six non-hashed molecular fingerprints (FPs),^24^ followed by the collection of relevant physicochemical properties and the calculation of six elemental mass defects (EMDs),^25^ predicted mobility classes,^22^ and logarithmic-scale ionization efficiency (LogIE) values.^26^ Full details are reported in Section S1 of the Supporting Information.

The chemical space is represented and analyzed by using PCA to identify regions for MCL sampling. Filters (see Section 2.2) were applied to select LC-HRMS-compatible candidates in both ionization modes.

The full workflow is depicted in Figure S1, including references to calculations and data availability.

### 2.2 Selection and Applicability of MCLs

Compound descriptors related to the CompTox dataset were obtained according to Sections S1.2 and S1.3 of the Supporting Information. PCA was conducted on 13 variables with the *ScikitLearn.jl* package, assigning numeric codes for mobility classification (i.e., “Non-mobile” = 1, “Mobile” = 2, and “Very mobile” = 3). Data were mean-centered and scaled before PCA for comparability. A symmetric gridding was applied to the dataset score plot to sample candidates across the selected chemical space (Table S1). Then candidates were extracted, retaining only the eligible structures according to the following criteria: (i) to avoid the inclusion of chemicals with high physicochemical and structural similarity, Jaccard distances were calculated using PCA variables to retain structures with distance values >0.15 (i.e., a Roger/Tanimoto score >0.85);^27^ (ii) to ensure the inclusion of ESI ionizable structures, the dataset was filtered according to LogIE >3.5 for positive ionization mode (ESI­(+)) and to LogIE <1.5 and hydrogen bond donor count >0 for negative ionization mode (ESI(−)). Since the LogIE prediction models were trained on LogIE values from ESI­(+) data, using a scale with methyl benzoate as anchor compound,^28^ for structures that are potentially ionizable in ESI(−), LogIE <1.5 was chosen as the threshold for lower probability of positive charge stabilization, also considering the presence of hydrogen bond donor functions that promote negative ionization. LogIE thresholds can be adjusted on (i) the initial dataset (chemical space) involved and (ii) the required ionization polarity.

Filtered structures were grouped by mobility class and then ranked to select a user-defined number of ESI­(+) candidates by descending LogIE, and ESI(−) candidates by descending LogIE <1.5.

The applicability of sampled MCLs was validated in terms of chemical coverage and retention behavior in comparison with the list of monitored CECs in the European Union water framework (Table S2).^14–16^ To assess chemical space coverage, besides PCA plots, the ClassyFire chemical taxonomy tool was used to identify the chemical class related to each structure (using InChIKeys) included in the MCLs.^29^ Retention behavior was investigated by means of retention index (RI) predictions using the *RIprediction.jl* package developed by van Herwerden et al.,^17^ providing supplementary retention classification related to RPLC subspace (i.e., −1 = “outside”, 0 = “maybe”, 1 = “inside” RPLC domain). After candidate selection, to assess the availability of purchasable standards inside the obtained MCLs, patent and literature counts for each structure were extracted using PubChem CIDs.

## 3 Results and Discussion

### 3.1 CompTox Space Distribution

PCA was performed to visualize the distribution of the CompTox chemical space (*n* = 785,294), as displayed in Figure S2. This dataset effectively balances the chemical subspace’s structural diversity with the known physicochemical distribution for a clear demonstration of the proposed approach.

The first three PCs explain about 84% of the variance in the original data (Figure S2A), representing the CompTox chemical subspace (Figure S2B). This spatial representation, now including mobility and LogIE predictions, shows no distinct trends, similar to Figure 1.

Based on explained variance, the loadings define a complex 3D space of structural and physicochemical features. PC1 scores/loadings decrease with molecular weight, XLogP, H-bonding features, and TPSA but increase with structure-related variables (mobility, LogIE, and EMDs) (Figure S2C). PC2 shows a partial reverse trend, with higher scores linked to greater polarity and molecular weight. In PC3, higher mobility corresponds to lower ionization efficiency, molecular weight, and XLogP. This is due to LogIE’s dependence on the positive ionization scale, which is reduced by electronegative groups that enhance molecular interactions and mobility.^30^

Mobility and LogIE variables indeed exhibit interesting patterns in the CompTox chemical space, as displayed by the PC spaces in Figures 2A and 2B. The distribution of “Non-mobile” structures is generally localized in a lower region (>molecular weight and XLogP) of the subspace, aligning with the increase of molecular weight and hydrophobicity,^20,31^ also suggesting the compatibility of this chemical space region with the reversed-phase chromatographic domain and high ESI­(+) IE.

**Figure 2:**
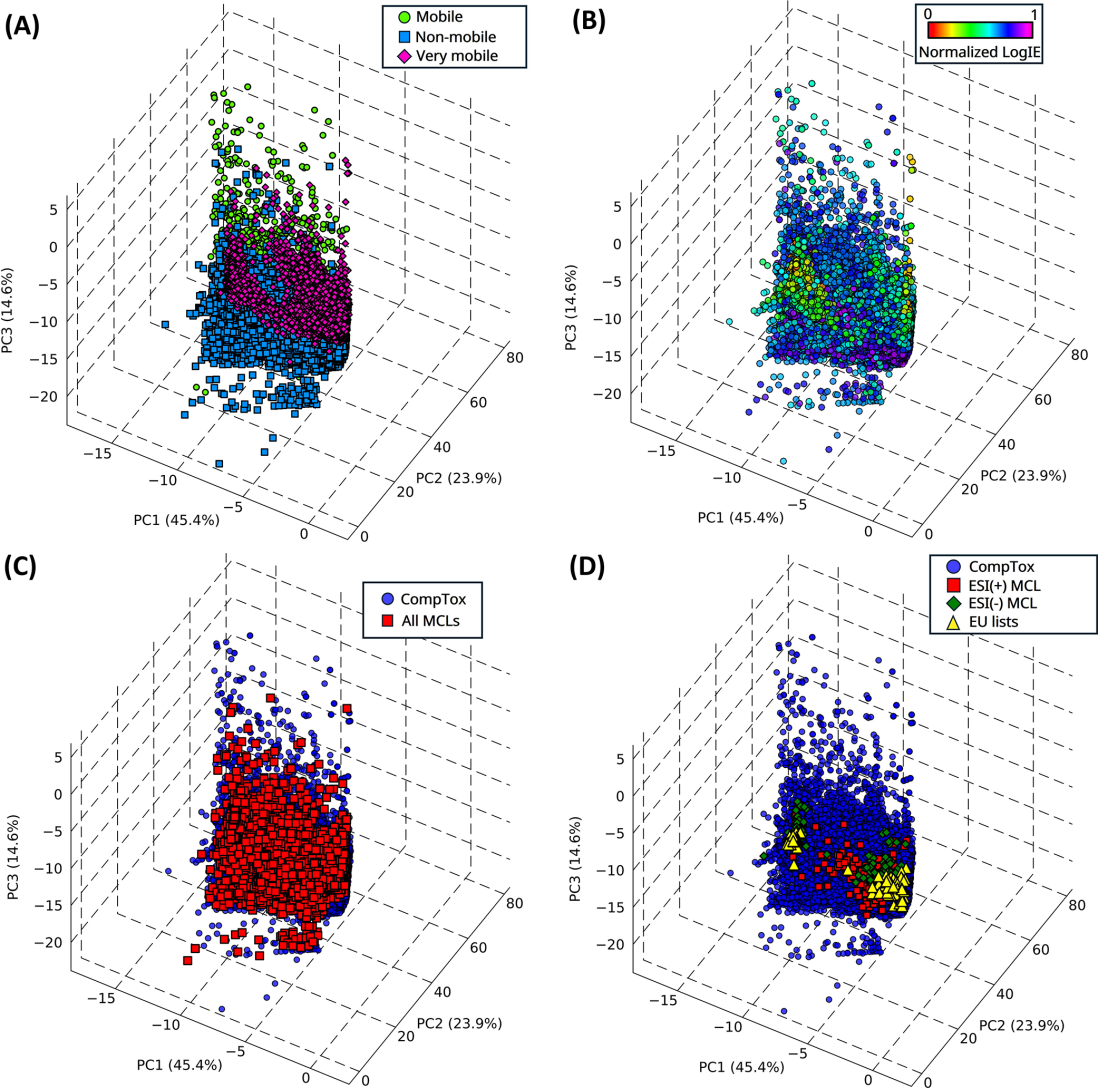
PCA score plots of CompTox structures (*n* = 785,294) highlighting (A) predicted mobility, (B) predicted ionization efficiency (LogIE normalized scale), (C) sampled chemicals for MCLs (red squares, *n* = 17,743), and (D) sampled candidates for ESI­(+) MCL (red squares, *n* = 150) and ESI(−) MCL (green diamonds, *n* = 150) in comparison with chemicals monitored under the EU water framework (Table S2). See Section S5 of the Supporting Information for grayscale plots.

Instead, LogIE seems to be negatively affected by the increase of mobility, as the ionizable moieties stabilizing positive charges gradually decrease with molecular weight, other than the increase of electronegative functions more prone to ionize in ESI(−).^32^ The presence of highly mobile structures suggests that the CompTox subspace includes chemicals beyond the reversed-phase LC domain, highlighting the need to consider additional selectivity mechanisms in NTA for its measurability. Including structures with varied mobility makes MCLs effective in defining measurable space boundaries under specific analytical conditions.

This complex scenario underlines the need for “data-driven” sampling, collecting representative structures for MCLs throughout the entire chemical subspace to preserve the original physicochemical variability involved in the CompTox database.

### 3.2 MCL Selection and Validation

Our approach aims to maximize the coverage of diverse structures out of the thousands encompassed in the chemical subspace, which was achieved through the symmetric gridding procedure (Table S1) and the use of Jaccard distances. Figure 2C shows the outcome of the MCL selection workflow, highlighting in the PC scores plot the selected structures (red squares, *n* = 17,743) that are homogeneously distributed in all the PC space according to the applied criteria, also reducing the risk of oversampling from high-density areas within the scores space compared to a pure random sampling. Additionally, the use of LogIE filters helped to retain only chemicals highly compatible with ESI­(+) and ESI(−), thus already highlighting a wide space of measurable LC-HRMS NTA chemicals. The ESI(−) compatibility criteria applied to the CompTox dataset included structures containing electronegative and resonance-stabilizing functions promoting a negative charge (see atomic FPs in the *Data preprocessed descriptors* at DOI: 10.6084/m9.figshare.28788143.v3). The use of ESI IE-based criteria restricts MCL sampling for apolar or neutral compounds more compatible with APCI, “biasing” MCL extended chemical coverage.^33,34^ However, in the CompTox space, the threshold LogIE <1.5 included structures like bile acids that are compatible with both ESI and APCI.

To better scale the selected structures for a feasible analytical standard selection, it is advisable to pick a specific number of candidate structures (e.g., *n* = 50) from each mobility category. These candidates are sorted in descending order based on the maximum LogIE values for ESI­(+) MCL and on LogIE values <1.5 for ESI(−) MCL (supplementary data available at DOI: 10.6084/m9.figshare.28788143.v3). The number of MCL candidates can be tuned depending on the selected chemical space and required chemical coverage.

#### 3.2.1 Chemical Coverage of MCLs

Figure 2D displays the result of the PC scores overlap by the ESI­(+) and ESI(−) MCLs within the CompTox chemical space and the CEC structures belonging to EU monitoring lists (Table S2), since there are no alternative approaches available for comparison. MCLs provided a greater coverage of the PC space compared to the EU-monitored chemicals, supporting the rationale behind the use of MCLs for LC-HRMS NTA method development.

The classification density distribution for MCL ESI­(+) and ESI(−) structures (*n* = 300) vs the EU CECs by the ClassyFire tool (see section 2.2) is summarized in Figure S3.

MCLs encompass a broader range of chemical classes than EU lists due to the uniform sampling of CompTox structures. While there is some overlap with EU CECs (e.g., piperazines and diphenylmethanes), the latter are skewed on specific classes of chemicals (e.g., alkyl fluorides), demonstrating how class-dependent internal standards can bias NTA method development and chemical coverage.

#### 3.2.2 Chromatographic and Mass Domain of MCLs

Alongside broad chemical coverage, MCLs need to reflect diverse retention behaviors, thanks to mobility class prediction trained on chromatographic data, including gradient and organic modifier content.^22^

Predicted RI values were calculated to demonstrate the range of the chromatographic domain coverage for the candidate structures of both ESI­(+) and ESI(−) MCLs. Figure 3 shows the results of the RI predictions based on the cocamide scale versus RPLC classes and monoisotopic masses from both MCLs and EU CECs. The sampled structures exhibited a wide RI coverage (5 < RI < 900), being nonetheless fully aligned with the coverage of EU chemicals “inside” the RPLC domain, with only a few exceptions for ESI(−) MCL candidates with RI ≈ 900 (Figure 3A). Among EU CECs, only metformin and guanylurea were classified as “maybe inside” and “outside” RPLC domains, whereas MCLs included several candidates in these alternative chromatographic domains, thus stepping outside the linearity assumption limiting comprehensive chemical measurements. Recalling the discussion in section 3.1, MCLs can assist NTA coverage under different LC conditions (e.g., hydrophilic interaction and supercritical fluid chromatography).^8,17^ Figure S4 supports this evidence, showing some of the MCL candidates with available experimental records (RepoRT database)^35^ in alternative chromatographic setups beyond RP C18, including phenyl and HILIC stationary phases.

**Figure 3:**
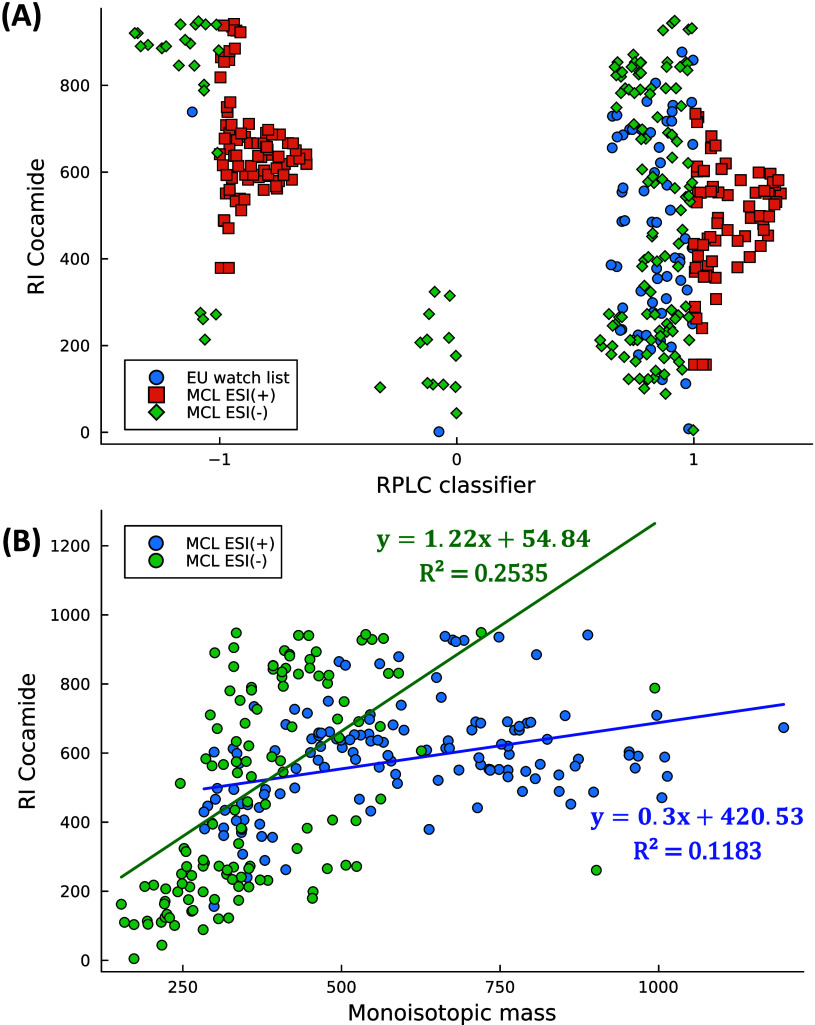
Predicted retention index (RI) values based on the cocamide scale for ESI(−) and ESI­(+) MCLs plotted against (A) retention classification related to RPLC subspace (−1 = “outside”, 0 = “maybe”, 1 = “inside”, vs EU monitored chemicals) and (B) monoisotopic mass. Linear regression trend lines and equations summarize the distinct characteristics of the two distributions.

Additionally, the MCL candidates have been analyzed for their coverage of the mass domain in relation to the predicted RIs (Figure 3B). The distribution of the monoisotopic masses of the combined MCLs covers the 100–1200 Da range. This outcome aligns with the PCA trend of molecular weights (section 3.1). Furthermore, the masses of the MCL structures exhibit weak linear trends when compared to RIs (*R* ^2^ ≈ 0.1–0.2), supporting our hypothesis that the reduced structure–retention relationships enhance the chemical coverage for NTA method development.

### 3.3 Availability of MCL Subset

A final consideration for applying the selected MCLs is the availability of the structures as reference standards for LC-HRMS analysis. Table S3 shows the results on the ESI­(+) and ESI(−) structures (*n* = 300) according to the patent and literature references extracted from the PubChem database. 72% (*n* = 217) of the selected structures possess records of patent registrations and/or literature citations, encouraging their potential availability as purchasable standards. These candidates preserved an optimal chemical space coverage of the PC score plot of the CompTox dataset (Figure S5), fully in line with the complete MCL lists (Figure 2D).

The availability of analytical standards depends on the user, method, and target chemical space. If a standard is unavailable or more candidates are needed, the PCA score matrix can guide the selection of alternative structures.

## 4 Environmental Implication

Sampling MCLs across a vast chemical (sub)­space of interest can strongly reduce the “biased coverage” in NTA method development, providing reliable internal standard lists stretching (beyond the RPLC linearity assumption) and defining the measurable chemical space under multiple LC-HRMS experimental conditions. Furthermore, it applies to any chemical subspace, from exposomics to metabolomics, with an available molecular representation.

Contextually, by utilizing key structural and physicochemical variables, such as mobility and ionization efficiency, MCL selection maintains the diversity of the CompTox space and yields lists compatible with LC-HRMS analysis in both ionization modes.

Moreover, MCL subsets based on mobility and ionization efficiency cover a significant range of the CompTox space regarding the mass range, predicted retention index, and structural variability, exceeding the chemical classes outlined in the European “watch lists” for water monitoring.

In the specific context of the exposome chemical subspace, represented here by the CompTox dataset, we consider MCLs to be effective tools for understanding and expanding the chemical coverage of NTA methods in identifying unknown or undetected CECs. MCLs not only have the potential to enhance the rate of CEC discovery but also can assist users in assessing the boundaries of chemical space, thereby reducing the risk of false positive detections in environmental analysis.

## Supplementary Material



## Data Availability

The input and output datasets retrieved in this study are available as .csv files and can be found at 10.6084/m9.figshare.28788143.v3. The code that was used to perform the calculations and MCL selection is available at https://bitbucket.org/laporen/mcl_selection_workflow/src/main/. The packages used for variable collection and prediction are also available: PubChemCrawler.jl - https://github.com/JuliaHealth/PubChemCrawler.jl; Mobility_prediction.jl - https://github.com/tobihul/Mobility_prediction; IE_prediction.jl - https://github.com/pockos56/IE_prediction.jl; and ClassyFireR - https://rdrr.io/cran/classyfireR/f/vignettes/Getting-Started.Rmd.

## Notes

S1, dataset creation; S2, principal component analysis of the CompTox dataset; S3, chemicals on European monitoring lists; S4, MCL selection and validation; and S5, appendix (PDF)
