# Supplementary material for: A Novel Chemical-Space-Dependent Strategy for Compound Selection in Non-target LC-HRMS Method Development Using Physicochemical and Structural Data
Source: Environ Sci Technol Lett. 2025 Aug 18;12(9):1162–8. doi: 10.1021/acs.estlett.5c00759 (PMC12424467; doi:10.1021/acs.estlett.5c00759)
Supplement: Supplementary file 1 [file ez5c00759_si_001.pdf]

A Novel Chemical Space Dependent Strategy for Compound Selection in Non-Target LC–HRMS Method Development Using Physicochemical and Structural Data.

Lapo Renai[a*], Viktoriia Turkina[a], Tobias Hulleman[b,c], Alexandros Nikolopoulos[a], Andrea F. G. Gargano[a], Elvio Amato[d], Massimo Del Bubba[e], Saer Samanipour[a,b,f**]

[a] *Van 't Hoff Institute for Molecular Sciences (HIMS), University of Amsterdam, 1090 GD, Amsterdam, the Netherlands*

[b] *Queensland Alliance for Environmental Health Sciences (QAEHS), 20 Cornwall Street, Woolloongabba, QLD, 4102, Australia*

[c] *ARC Training Centre for Hyphenated Analytical Separation Technologies (HyTECH), Queensland Alliance for Environmental Health Sciences (QAEHS), 20 Cornwall Street, Woolloongabba, QLD, 4102, Australia*

[d] *KWR Water Research Institute, Groningenhaven 7 3433 PE Nieuwegein, Netherlands*

[e] *Department of Chemistry, University of Florence, Via della Lastruccia 3, 50019, Sesto Fiorentino, Florence, Italy*

[f] *UvA Data Science Center, University of Amsterdam, 1012 WP, Amsterdam, the Netherlands.*

*Corresponding author.

**Corresponding author. Van 't Hoff Institute for Molecular Sciences (HIMS), University of Amsterdam, Amsterdam, 1098 XH, the Netherlands.

E-mail addresses: l.renai@uva.nl (L. Renai), s.samanipour@uva.nl (S. Samanipour).

Contents

S1. Dataset creation 3

S1.1 CompTox database and fingerprints calculation 3

S1.2 PubChem descriptors and EMD calculations 3

S1.3 Mobility and ionization efficiency predictions 3

S2. Principal component analysis of the CompTox dataset 5

S2.1 Principal component analysis outputs 5

S2.2 Symmetric gridding 5

S3. Chemicals of European monitoring lists 6

S4. MCL selection & validation 7

S4.1 Chemical coverage of sampled MCLs 7

S4.2 Chromatographic domain of MCLs 8

S4.3 Standard availability of MCL candidates 8

S5. Appendix 9

S1. Dataset creation

S1.1 CompTox database and fingerprints calculation

About 858k CompTox chemical list containing SMILES and InChiKeys were downloaded from the CompTox dashboard (https://comptox.epa.gov/dashboard/chemical-lists, accession date: March 2024).[1] Canonical SMILES were used to calculate six non-hashed fingerprints (FPs),[2] computed using the PaDEL software package implemented via a Python 3 wrapper called PaDELPy (version 0.1.13),[3] with the exception of E-state FPs, calculated using the RDKit software package (version 2022.9.5).[4] Finally, full structural and descriptor data were stored in separate *.csv* files (about 1000 rows each). The computed FPs are used for mobility and ionization predictions (paragraph 2.2.3).

S1.2 PubChem descriptors and EMD calculations

The following structural and physicochemical descriptors were sourced from the PubChem database (https://pubchem.ncbi.nlm.nih.gov/) using the *PubChemCrawler.jl* package using the valid CID from each structure listed in the dataset: InChIKey, exact mass, molecular weight, XLogP, hydrogen bond donor count, hydrogen bond acceptor count, and total polarizable surface area (TPSA). To increase the information on the chemical space coverage, elemental mass defects (EMDs) of six elemental ratios (i.e., CO, CCl, CN, CS, CF, and CH) were calculated as elsewhere reported for each structure included in the dataset.[5] Similarly, literature and patent references were sourced from PubChem database as metrics for analytical standard availability for the final list of MCL candidates.

S1.3 Mobility and ionization efficiency predictions

The mobility random forest (RF) classifier model developed by Hulleman et al.[6] was used to predict the three mobility classes for all the CompTox structures, defined as “Very mobile”, “Mobile”, and “Non-mobile”.[7] Similarly, predicted logIE values were calculated using the tool developed by Nikolopoulos et al.,[8] setting the pH value to 7 to average the speciation of functional groups occurring in the dataset structures.

Alternatively to the former calculation of FPs, it is possible to use both prediction tools starting from canonical SMILES, at the expense of the speed depending on the number of structures to be iterated.

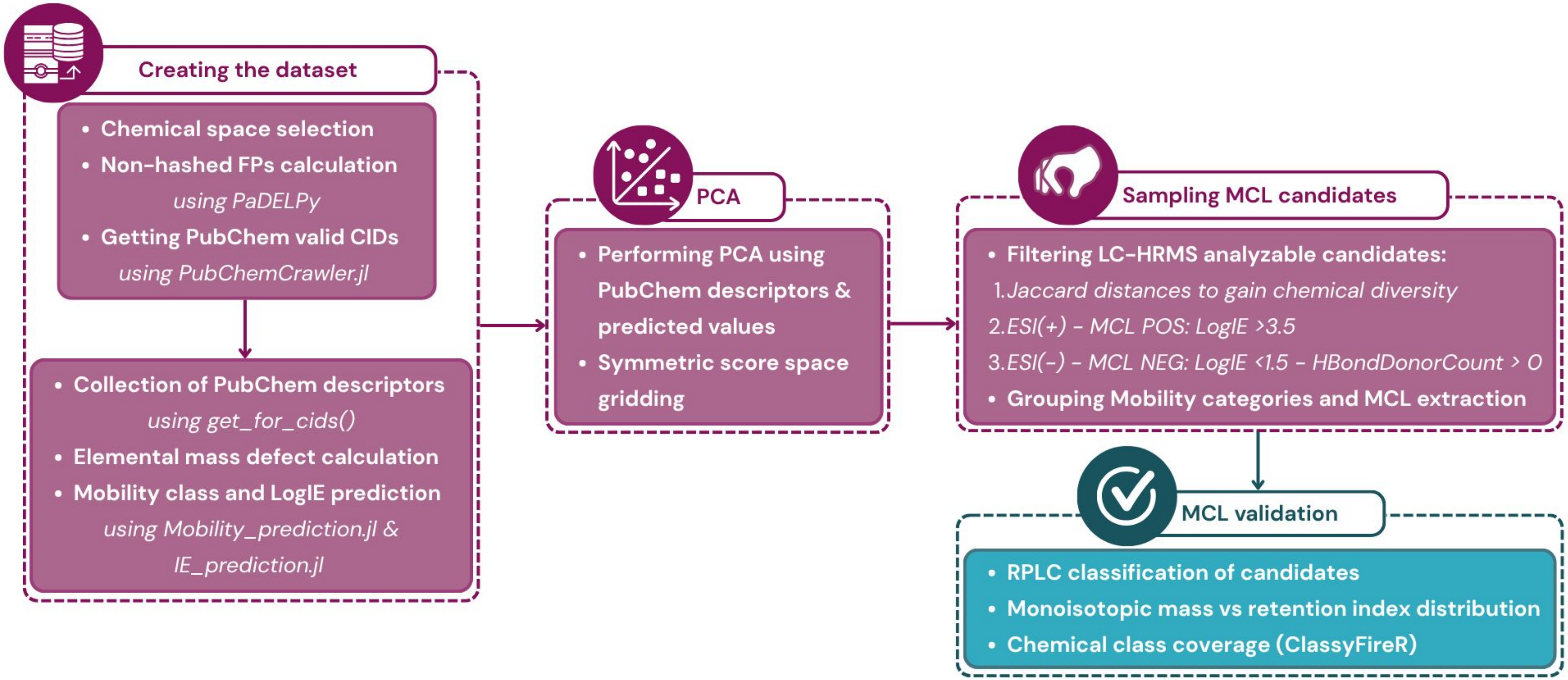

Figure S1 – Schematic representation of the presented data-driven approach: from dataset creation to MCL selection and validation.
All calculations were performed on a personal computer (PC) with an Intel Core i9-14900HX central processing unit and 32 GB of RAM operating Windows 11 Education version 23H2. All of the data processing and statistical analyses were performed using Julia language version 1.11.2, with the exception of chemical classification by ClassyFire which was run using R language version 4.4.2.
Code for dataset creation and MCL selection can be found at https://bitbucket.org/laporen/mcl_selection_workflow/src/main/.
Data are available at https://doi.org/10.6084/m9.figshare.28788143.v3.

S2. Principal component analysis of the CompTox dataset

S2.1 Principal component analysis outputs

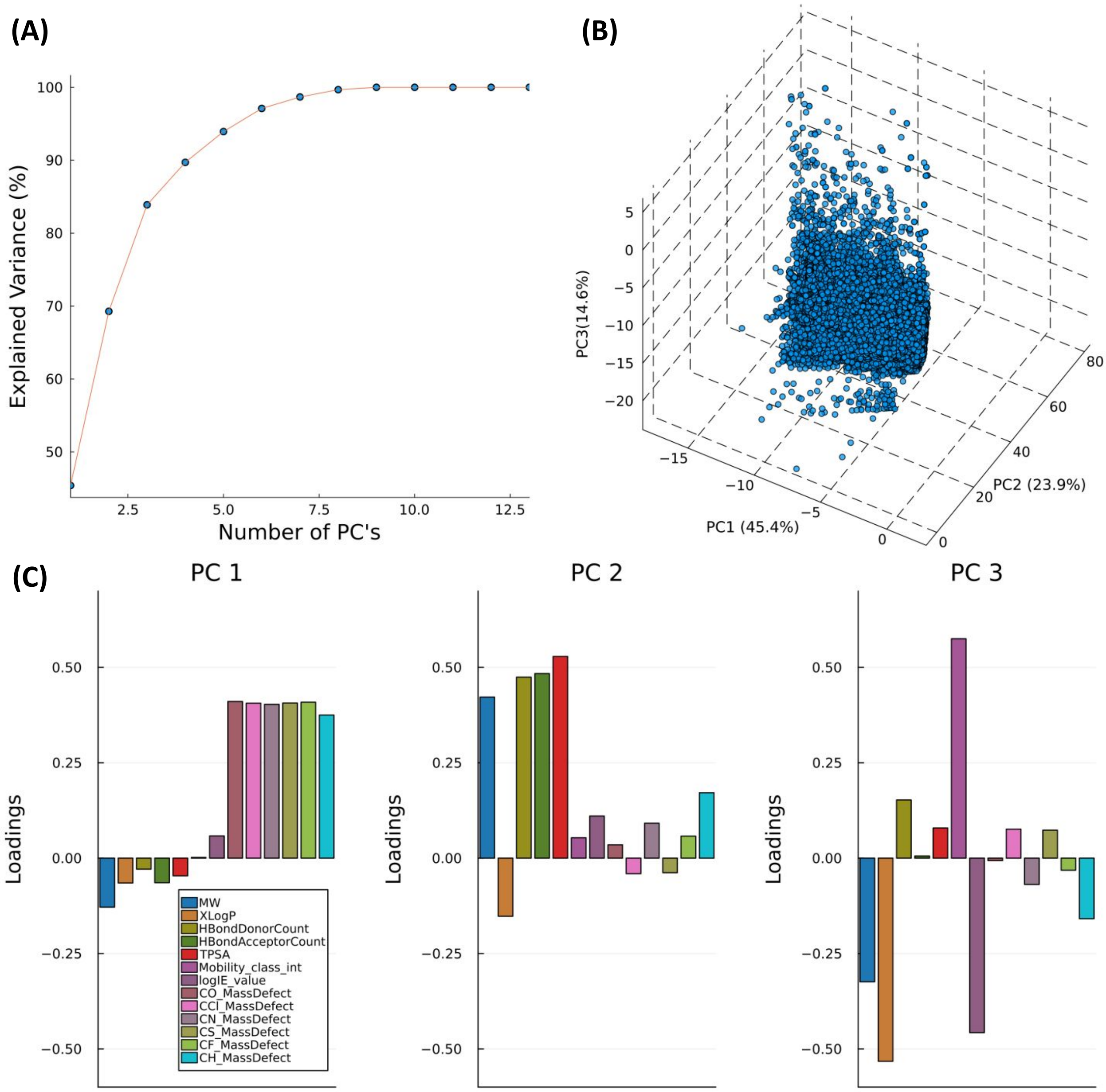

Figure S2 – Outputs of the principal component analysis on the CompTox dataset (n=785,294 structures). (A) Plot of the explained variance (%) for the calculated principal components (PCs). (B) Score plot of the first 3 components. (C) Histograms of the loadings of the original variables on the first 3 components.

S2.2 Symmetric gridding

Table S1 – Symmetric gridding rationalized for the CompTox PC score space.

PC component	Lower limit	Upper limit	Step
PC1	-15	0	7.5
PC2	0	60	30
PC3	-20	5	12.5

S3. Chemicals of European monitoring lists

Table S2 – 62 chemicals included in the EU water monitoring framework and listed according to compound name, PubChem identifier (CID), InChiKey, and monoistopic mass.

Compound name	CID	InChIKey	Monoisotopic Mass
Sulfamethoxazole	5329	JLKIGFTWXXRPMT-UHFFFAOYSA-N	253.0521
Trimethoprim	5578	IEDVJHCEMCRBQM-UHFFFAOYSA-N	290.1379
Venlafaxine	5656	PNVNVHUZROJLTJ-UHFFFAOYSA-N	277.2042
O-desmethylvenlafaxine	125017	KYYIDSXMWOZKMP-UHFFFAOYSA-N	263.1885
Clotrimazole	2812	VNFPBHJOKIVQEB-UHFFFAOYSA-N	344.1080
Itraconazole	55283	VHVPQPYKVGDNFY-ZPGVKDDISA-N	704.2393
Imazalil	37175	PZBPKYOVPCNPJY-UHFFFAOYSA-N	296.0483
Temocillin	171758	BVCKFLJARNKCSS-DWPRYXJFSA-N	414.0555
Metconazole	86210	XWPZUHJBOLQNMN-UHFFFAOYSA-N	319.1451
Miconazole	4189	BYBLEWFAAKGYCD-UHFFFAOYSA-N	413.9860
Penconazole	91693	WKBPZYKAUNRMKP-UHFFFAOYSA-N	283.0643
Prochloraz	73665	TVLSRXXIMLFWEO-UHFFFAOYSA-N	375.0308
Tebuconazole	86102	PXMNMQRDXWABCY-UHFFFAOYSA-N	307.1451
Tetraconazole	80277	LQDARGUHUSPFNL-UHFFFAOYSA-N	371.0215
Dimoxystrobin	10936292	WXUZAHCNPWONDH-DYTRJAOYSA-N	326.1630
Azoxystrobin	3034285	WFDXOXNFNRHQEC-GHRIWEEISA-N	403.1168
Famoxadone	213032	PCCSBWNGDMYFCW-UHFFFAOYSA-N	374.1267
Diflufenican	91735	WYEHFWKAOXOVJD-UHFFFAOYSA-N	394.0741
Fipronil	3352	ZOCSXAVNDGMNBV-UHFFFAOYSA-N	435.9387
Clindamycin	446598	KDLRVYYVGXIQJDK-AWPVFWJPSA-N	424.1799
Ofloxacin	4583	GSDSWSVVBLHKDQ-UHFFFAOYSA-N	361.1438
Metformin	4091	XZWYZXLIPXDOLR-UHFFFAOYSA-N	129.1014
Guanylurea	8859	SQSPRWMERUQXNE-UHFFFAOYSA-N	102.0542
Octocrylene	22571	FMJSMJQBSVNSBF-UHFFFAOYSA-N	361.2042
Benzophenone-3	4632	DXGLGDHPHMLXJC-UHFFFAOYSA-N	228.0786
Amisulpride	2159	NTJOBXMMWNYJFB-UHFFFAOYSA-N	369.1722
Carbamazepine	2554	FFGPTBGBLSHEPO-UHFFFAOYSA-N	236.0950
Citalopram	2771	WSEQXVZVJXJVFP-UHFFFAOYSA-N	324.1638
Clarithromycin	84029	AGOYDEPGAOXOCK-KCBOHYOISA-N	747.4769
Diclofenac	3033	DCOPUUMXTXDBNB-UHFFFAOYSA-N	295.0167
Hydrochlorothiazide	3639	JZUFKLXOESDKRF-UHFFFAOYSA-N	296.9645
Metoprolol	4171	IUBSYMUCCVWXPE-UHFFFAOYSA-N	267.1834
Benzotriazole	7220	QRUDEWIWKLJBPS-UHFFFAOYSA-N	119.0483
Candesartan	2541	HTQMVQVXFRQIKW-UHFFFAOYSA-N	440.1597
Irbesartan	3749	YOSHYTLCDANDAN-UHFFFAOYSA-N	428.2325
4-Methylbenzotriazole	122499	CMGDVUCDZOBDNL-UHFFFAOYSA-N	133.0640
5-methylbenzotriazole	8705	LRUDIIUSNGCQKF-UHFFFAOYSA-N	133.0640
Bisphenol A	6623	IISBACLAFKSPIT-UHFFFAOYSA-N	228.1150
Perfluoropropanoic acid	62356	LRMSQVBRUNSOJL-UHFFFAOYSA-N	163.9897
Trifluoroacetic acid	6422	DTQVDTLACAAQTR-UHFFFAOYSA-N	113.9929
Perfluorohexanoic acid	67542	PXUULQAPEKKVAH-UHFFFAOYSA-N	313.9801
Perfluoroheptanoic acid	67818	ZWBAMYVPMDSJGQ-UHFFFAOYSA-N	363.9769
Perfluorooctanoic acid	9554	SNGREZUHAYWORS-UHFFFAOYSA-N	413.9737
Perfluorononanoic acid	67821	UZUFPBIDKMEQEQ-UHFFFAOYSA-N	463.9705
Perfluorodecanoic acid	9555	PCIUEQPBYFRTEM-UHFFFAOYSA-N	513.9673
Perfluoroundecanoic acid	77222	SIDINRCMMRKXGQ-UHFFFAOYSA-N	563.9641
Perfluorododecanoic acid	67545	CXGONMQFMIYUJR-UHFFFAOYSA-N	613.9609
Perfluorotridecanoic acid	3018355	LVDGGZAZAYHXEY-UHFFFAOYSA-N	663.9577
Perfluorotetradecanoic acid	67822	RUDINRUXCKIXAJ-UHFFFAOYSA-N	713.9545
N-Methylperfluorooctane Sulfonamide	3034468	SRMWNTGHXHOWBT-UHFFFAOYSA-N	570.9746
N-Ethylperfluorooctane sulfonamide	77797	CCEKAJIANROZEO-UHFFFAOYSA-N	584.9903
Perfluorobutanoic acid	9777	YPJUNDFVDDCYIH-UHFFFAOYSA-N	213.9865
Perfluoropentanoic acid	75921	CXZGQIAOTKWCDB-UHFFFAOYSA-N	263.9833
Perfluorobutanesulfonic acid	67815	JGTNAGYHADQMCM-UHFFFAOYSA-N	299.9503
Perfluoropentanesulfonic acid	75922	ACEKLXZRZOWKRY-UHFFFAOYSA-N	349.9471
Perfluorohexanesulfonic acid	67734	QZHDEAJFRJCDMF-UHFFFAOYSA-N	399.9439
Perfluoroheptanesulfonic acid	67820	OYGQVDSRYXATEL-UHFFFAOYSA-N	449.9407
Perfluorooctanesulfonic acid	74483	YFSUTJLHUFNCNZ-UHFFFAOYSA-N	499.9375
Perfluorononanesulfonic acid	86998	MNEXVZFQQPKDHC-UHFFFAOYSA-N	549.9343
Perfluorodecanesulfonic acid	67636	HYWZIAVPBSTISZ-UHFFFAOYSA-N	599.9311
Perfluorooctane sulfonamide	69785	RRRXPPIDPYTNJG-UHFFFAOYSA-N	498.9535

S4. MCL selection & validation

S4.1 Chemical coverage of sampled MCLs

Figure S3 – Chemical category density distribution of the candidate structures included in MCLs (purple) vs EU monitoring list chemicals (yellow) according to ClassyFire chemical taxonomy. Density was calculated normalizing the number of structures for each category for the total number included in each dataset.

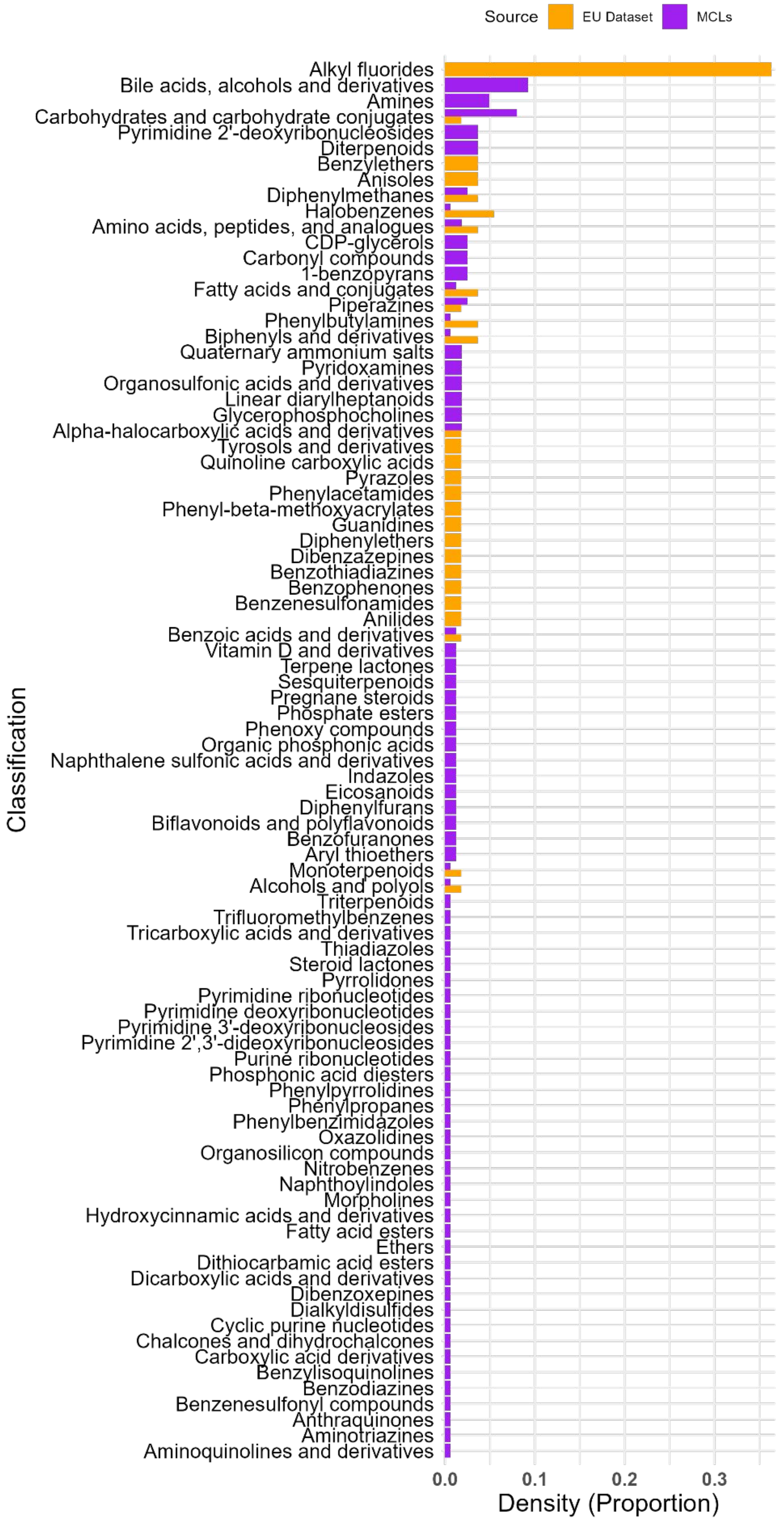

S4.2 Chromatographic domain of MCLs

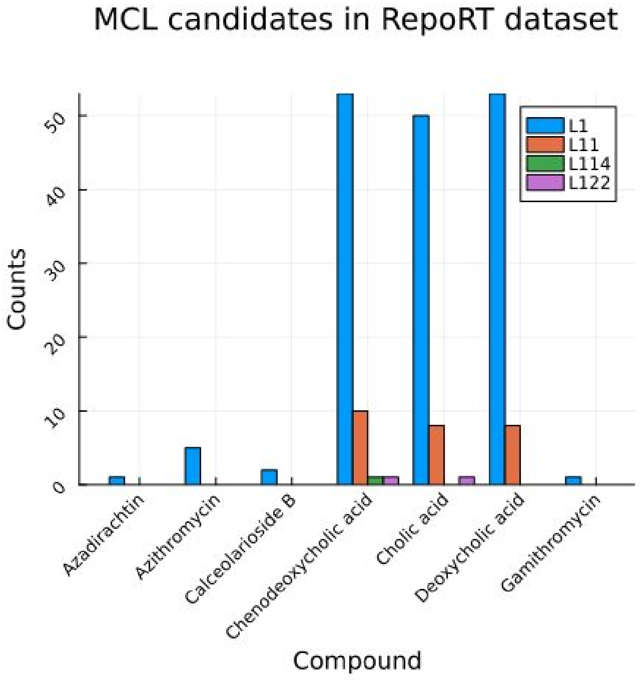

Figure S4 – Frequency of occurrence (counts) of MCL candidates in the RepoRT database (n = 437)[9] grouped according to stationary phase type (USP column codes). L1 = C18, L11 = phenyl, L114-L122 = sulfobetaine hydrophilic polymer.

S4.3 Standard availability of MCL candidates

Table S3 – Number of MCL candidates for which analytical standards are available according to patent and literature references from PubChem database (https://pubchem.ncbi.nlm.nih.gov/).

	MCL candidates
Patent	153
Literature	153
Patent & Literature	93
Available*	217
Not available**	83

*number of structures with highly probable analytical standard availability
**number of structures with no patent or literature reference

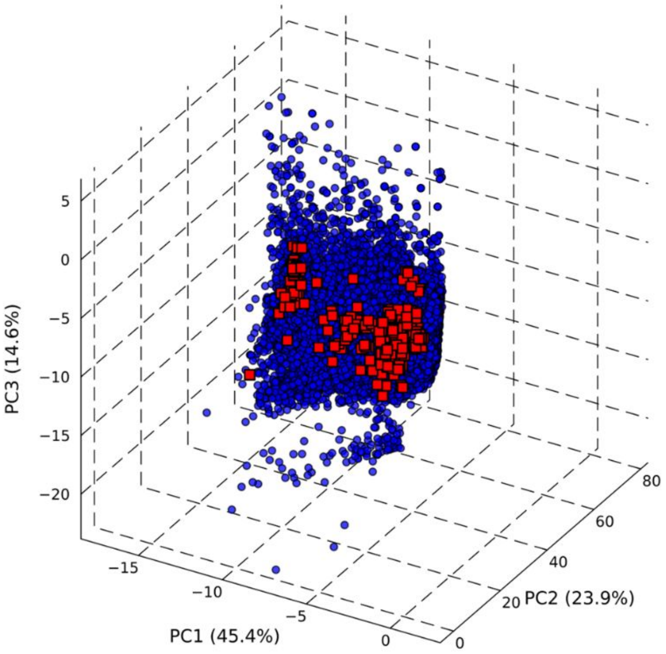

Figure S5 – PCA score plots of CompTox structures (n=785,294) highlighting the selected MCL candidates available as reference standards (red squares, n=217).

S5. Appendix

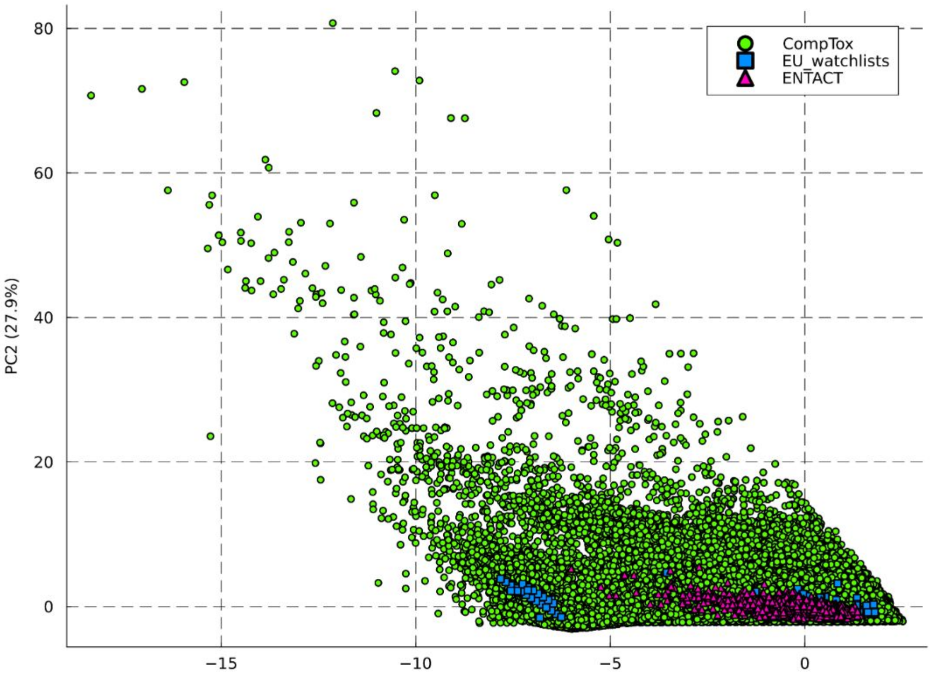

Figure S6 – [Referred to Figure 1] 2D Score plot of the principal component analysis (PCA) on chemical structures within CompTox database (n ~ 800k) and the overlap with the shared compounds listed in European water monitoring program (n = 62) and the prioritized list of the ENTACT initiative (n = 1019).

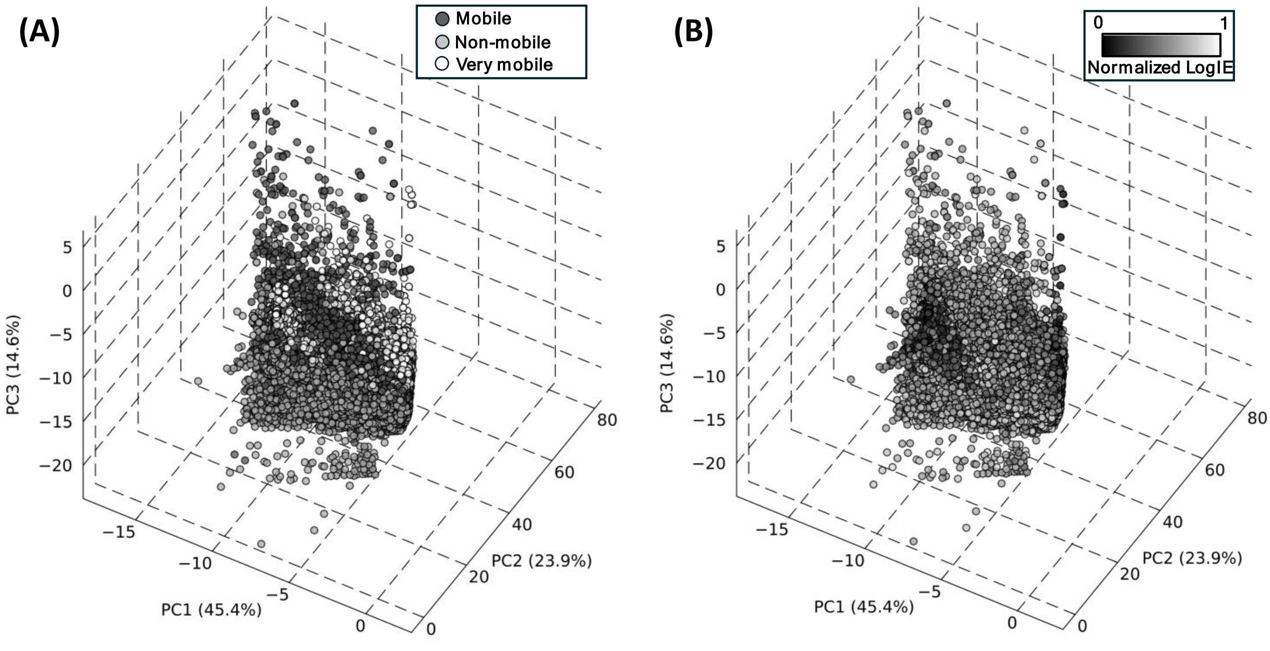

Figure S7 – [Referred to Figure 2] Grayscale PCA score plots of CompTox structures (n=785,294) highlighting (A) predicted mobility, (B) predicted ionization efficiency (LogIE normalized scale).

References

(1) Williams, A. J.; Grulke, C. M.; Edwards, J.; McEachran, A. D.; Mansouri, K.; Baker, N. C.; Patlewicz, G.; Shah, I.; Wambaugh, J. F.; Judson, R. S.; Richard, A. M. The CompTox Chemistry Dashboard: A Community Data Resource for Environmental Chemistry. *J Cheminform* **2017**, *9* (1). https://doi.org/10.1186/s13321-017-0247-6.

(2) Turkina, V.; Messih, M. R. W.; Kant, E.; Gringhuis, J.; Petrignani, A.; Corthals, G.; O'Brien, J. W.; Samanipour, S. Molecular Fingerprints Optimization for Enhanced Predictive Modeling. February 26, 2024. https://doi.org/10.26434/chemrxiv-2024-zr2vr.

(3) Yap, C. W. PaDEL-Descriptor: An Open Source Software to Calculate Molecular Descriptors and Fingerprints. *J Comput Chem* **2011**, *32* (7), 1466–1474. https://doi.org/10.1002/jcc.21707.

(4) Bento, A. P.; Hersey, A.; Félix, E.; Landrum, G.; Gaulton, A.; Atkinson, F.; Bellis, L. J.; De Veij, M.; Leach, A. R. An Open Source Chemical Structure Curation Pipeline Using RDKit. *J Cheminform* **2020**, *12* (1). https://doi.org/10.1186/s13321-020-00456-1.

(5) Sleno, L. The Use of Mass Defect in Modern Mass Spectrometry. *Journal of Mass Spectrometry* **2012**, *47* (2), 226–236. https://doi.org/10.1002/jms.2953.

(6) Hulleman, T.; Samanipour, S.; Haddad, P. R.; Rauert, C.; Okoffo, E. D.; Thomas, K. V; O'brien, J. W. *Machine Learning for Predicting Environmental Mobility Based on Retention Behaviour*; **2025**. https://doi.org/https://doi.org/10.26434/chemrxiv-2025-xl6xl.

(7) Katrina White. *Guidance for Reporting on the Environmental Fate and Transport of the Stressors of Concern in Problem Formulations for Registration Review, Registration Review Risk Assessments,*

Listed Species Litigation Assessments, New Chemical Risk Assessments, and Other Relevant Risk Assessments.

(8) Nikolopoulos, A.; van Herwerden, D.; Turkina, V.; Kruve, A.; Baerenfaenger, M.; Samanipour, S. Ionization Efficiency Prediction of Electrospray Ionization Mass Spectrometry Analytes Based on Molecular Fingerprints and Cumulative Neutral Losses. **2025**. https://doi.org/10.26434/chemrxiv-2025-dc9gd.

(9) Kretschmer, F.; Harrieder, E. M.; Hoffmann, M. A.; Böcker, S.; Witting, M. RepoRT: A Comprehensive Repository for Small Molecule Retention Times. *Nature Methods*. Nature Research February 1, **2024**, pp 153–155. https://doi.org/10.1038/s41592-023-02143-z.
